# Urinary Catheterization in Medical Wards

**DOI:** 10.4103/0974-777X.62870

**Published:** 2010

**Authors:** Nirmanmoh Bhatia, Mradul K Daga, Sandeep Garg, S K Prakash

**Affiliations:** *Maulana Azad Medical College, New Delhi, India*; 1 *Department of Medicine, Maulana Azad Medical College, New Delhi, India*; 2 *Department of Microbiology, Maulana Azad Medical College, New Delhi, India*

**Keywords:** Bacterial colonization on Foley's catheters, Catheter associated urinary tract infections, Inappropriate catheterization, Medical wards

## Abstract

**Aims:**

The study aims to determine the: 1. frequency of inappropriate catheterization in medical wards and the reasons for doing it. 2. various risk factors associated with inappropriate catheterization, catheter associated urinary tract infections (CAUTI) and bacterial colonization on Foley's catheters (BCFC).

**Settings and Design:**

Hospital-based prospective study.

**Materials and Methods:**

One hundred and twenty five patients admitted consecutively in the medical wards of a tertiary care hospital, who underwent catheterization with a Foley's catheter, at admission, have been included in the study. Patient profiles were evaluated using the following parameters: age, sex, diagnosis, functional status, mental status, indication, duration and place of catheterization, development of BCFC and CAUTI.

**Statistical tests used:**

Chi-square test.

**Results:**

Thirty-six out of 125 (28.8%) patients included were inappropriately catheterized. BCFC developed in 52.8% and 22.4% were diagnosed with a CAUTI. The most frequent indication for inappropriate catheterization was urinary incontinence without significant skin breakdown (27.8%). The risk factors for inappropriate catheterization were female sex (RR=1.29, 95% CI=0.99, 1.69, *P*<0.05) and catheterization in the emergency (RR=0.74, 95% CI=0.61, 0.90, *P*<0.05). The risk factors for developing a BCFC were age>60 years (RR=0.65, 95% CI=0.48, 0.89, *P*<0.05), non-ambulatory functional status (RR=0.57, 95% CI=0.39, 0.84, *P*<0.01), catheterization in the emergency (RR=2.01, 95% CI=1.17, 3.46, *P*<0.01) and duration of catheterization>3 days (RR=0.62, 95% CI=0.43, 0.89, *P*<0.01). The risk factors for acquiring a CAUTI were age>60 years (RR=0.47, 95% CI=0.25, 0.90, *P*<0.05), impaired mental status (RR=0.37, 95% CI=0.18, 0.77, *P*<0.01) and duration of catheterization>3 days (RR=0.24, 95% CI=0.10, 0.58, *P*<0.01).

**Conclusions:**

Inappropriate catheterization is highly prevalent in medical wards, especially in patients with urinary incontinence. The patients catheterized in the medical emergency and female patients in particular are at high risk. Careful attention to these factors can reduce the frequency of inappropriate catheterization and unnecessary morbidity.

## INTRODUCTION

Indwelling urinary tract catheterization (IUTC) is a very common intervention frequently required in hospitalized patients. It is estimated that 10-12% of hospital patients and four per cent of patients in the community have urinary catheters *in situ* at any given time.[1] Nosocomial UTIs (urinary tract infections) develop in five per cent of catheterized patients per day in the US, with associated bacteremia in four per cent[2] and as many as 80% are a consequence of urinary catheters.[3] Fever, pyelonephritis, urinary tract stones and chronic renal inflammation are some of the other complications of this procedure.[4] IUTC also prolongs hospital stay and increases the cost of healthcare.[5] Unfortunately, inappropriate and excessive catheter use still persists.[6] Research has shown that just reminding physicians to remove unnecessary urinary catheters can significantly reduce the duration of urinary catheterization and the catheter associated urinary tract infection (CAUTI) rate in a hospital.[7]

It is generally not recommended to treat asymptomatic catheter associated bacteriuria.[8,9] However, it has been shown to be an important cause of hospital acquired urinary tract infections especially in post-operative patients.[10] Bacterial colonization on Foley's (urethral) catheters (BCFC) can precede the emergence of bacteriuria and has a significantly higher rate of culture positives as compared to urine culture.[11] This is especially true in the initial two to three days of catheterization. Hence, it is important to consider this parameter instead of a urine culture in order to obtain a more precise picture of asymptomatic infections of the urinary tract in catheterized patients.

One of the important reasons for inappropriate catheterization could be the lack of widely accepted guidelines regarding the indications for IUTC placement in medical patients. This makes the distinction between appropriate and inappropriate catheterization obscure. There are a limited number of studies, which have determined the appropriateness of IUTC use in medical patients admitted to hospitals. The reasons for inappropriate IUTC placement have not been adequately explored in this group. Similarly, the appropriateness of catheterization in the emergency setting and its association with the development of BCFC and CAUTI has not been investigated in previous studies.

We decided to conduct this study as no data is available for Indian patients. No previous study has attempted to investigate risk factors associated with BCFC. This is important because even though the significance of catheter-colonizing bacteria in the pathogenesis of urinary tract infection remains unclear, they are still involved in the development of catheter-associated urinary tract infection refractory to antimicrobial chemotherapy.[11] Therefore BCFC represents a potential source of CAUTI.

The primary aim of our study was to analyze the various indications for urinary tract catheterization in medical patients and determine the frequency of its inappropriate use. We have also attempted to investigate the development of BCFC and CAUTI as well as to determine the associated risk factors for these entities. This was done with the aim of devising effective infection control strategies for IUTC relevant to our setting, which may reduce the burden of nosocomial infections.

## MATERIALS AND METHODS

This study was conducted in a tertiary care referral hospital in New Delhi. One hundred and twenty five consecutive patients of either sex, admitted to the medical wards either directly or via the emergency with IUTC (Foley's catheters), were included in the study. Patients with condom catheters, suprapubic catheters and percutaneous nephrostomy tubes were not included in the study. Those catheterized prior to admission were also excluded. A baseline urinary culture was obtained for all included patients in order to exclude those with a pre-existing urinary tract infection. During the study period of two months, 1891 patients were admitted to the medical wards. Of these, 186 patients had some form of urinary catheter in place. Twenty-three patients were excluded due to a positive urine culture. A further 38 patients were excluded as they were not catheterized with a Foley's catheter.

An independent observer (the first author of the study) visited the medical wards every day and obtained the list of all admitted patients from the nurse on duty. Thereafter, he evaluated all these patients for enrolment in the study if they fulfilled the inclusion criteria. The observer evaluated the detailed clinical profile of all enrolled patients. These patients were followed up during his daily visits to the wards, till 48 hours after their catheters were removed. This was carried out in order to diagnose a CAUTI, if it developed. The catheter tip of all the patients included in the study was sent for culture to detect bacterial colonization. This was done irrespective of patients developing a symptomatic UTI during the course of their hospital stay. The intraluminal catheter surface was swabbed to prepare a suspension. The suspension was cultured with the dip slide method and the microorganisms identified.

A urine culture was sent by the observer in cases where patients developed symptoms suggestive of a UTI during the course of their follow up till 48 hours after removal of the catheters, in order to diagnose a CAUTI according to the CDC guidelines.

Data was collected by interviewing the treating physicians and from the patient's files. The physicians were asked the following questions: the place of catheterization and the indication for catheterization. Demographic data i.e. age and sex as well as diagnosis was obtained from the patient files. The results of the urine culture and catheter-tip culture were also obtained. The observer then evaluated their functional status and mental status.

Data was recorded for each patient using a proforma, which included the following parameters: age, sex, diagnosis at admission, functional status, mental status, indication for catheterization, place of catheterization (medical ward/emergency), duration of catheterization, development of a UTI during hospital stay, analysis of urine culture and catheter tip culture. Age was classified as >60 years and <60 years, functional status was classified as ambulatory and non-ambulatory (this was a subjective assessment of the observer), mental status was classified as alert or impaired (confused, drowsy, stuporous, and comatose).[12,13] The indications for catheterization were classified as appropriate and inappropriate based on previously published acceptable indications for catheterization.[14–16] The appropriate indications are shown in Table 1. All other indications were classified as inappropriate.

**Table 1:** Appropriate indications for use of an indwelling urinary tract catheter

1.	Obstruction to the urinary tract distal to bladder
2.	Need to measure urine output accurately in an uncooperative patient (e.g., intoxication)
3.	Fluid challenge in patients with acute renal insufficiency
4.	Preoperative catheter insertion for patients going directly to the operation room
5.	Urinary incontinence posing a risk to patient (e.g., major skin breakdown, protection of nearby operative site or prone to infections)
6.	Trauma
7.	Paralysis (hemiplegia, paraplegia etc.)
8.	Acute urinary retention
9.	Patients with neurogenic bladder or retention
10.	Surgery on contiguous structures
11.	Urologic surgery
12.	Critically ill or postoperative patients requiring accurate measure of urinary output
13.	Palliative care for termi nally ill or severely impaired incontinent patients for whom bed and clothing changes are uncomfortable

In cases where no explicit indication for IUTC placement could be determined, the authors analyzed the clinical scenario and determined the appropriateness. For example, patients who had an ‘altered sensorium’ due to CNS (central nervous system) involvement were considered appropriate candidates for catheterization as they can be incontinent and are at subsequent high risk of infections in the absence of urinary catheterization. (See criterion number 5 in Table 1) These patients also require accurate monitoring of urine output, in order to supplement the correct amount of fluid. (See criterion 12 in Table 1).

To further verify the accuracy of the determination of the presence or absence of an appropriate indication for catheter placement in all the patients in the study, three authors (first, second and third author of the study) independently reviewed the cases of 25 randomly selected patients out of the 125 patients, who were included in the study. There was complete agreement in all these 25 cases.

Data was analyzed using WHO's Epi-Info 2005. Inter-relationships between various variables, the statistical significance of various risk factors for inappropriate catheterization, development of a BCFC, CAUTI and duration of catheterization were determined by using appropriate statistical methods.

## RESULTS

Out of 125 patients with an IUTC included in the study, 80 were males and 45 females (ratio: 1.78:1). Their age ranged from 15 to 86 years with 81/125 (64.8%) older than 60 years. Fifty-four (43.2%) patients were ambulatory. Only 33/125 (26.4%) patients were catheterized in the wards and the rest 92/125 (73.6%) in the medical emergency. Sixty (48%) patients' mental status was classified as impaired.

The mean duration of catheterization in the included patients was 4.8 days with a range from one day (minimum) to 16 days (maximum). The mean duration of catheterization for patients catheterized in the emergency was 4.3 days versus 6.2 days for those catheterized in the wards. (*P*<0.01)

Majority of patients (64%) had a CNS (42/125, 33.6%) or a GI (gastro-intestinal) (38/125, 30.4%) system involvement at diagnosis. Most patients with inappropriate catheterization were also from these two groups (CNS – 16/42 and GI - 8/38) as shown in Table 2.

**Table 2:** Diagnoses at admission with frequency of inappropriate catheterization in each group

Admitting diagnoses	Number of patients	Number of patients with inappropriate catheterization
Nervous system	42	16 (38.1%)
Cardiovascular system	15	4 (26.7%)
Genitourinary system	8	2 (25%)
Respiratory system	11	2 (18.2%)
Gastrointestinal system	35	8 (22.9%)
Other	14	4 (28.6%)

### Catheter associated urinary tract infections and bacterial colonization on Foley's catheters

**Figure 1:**
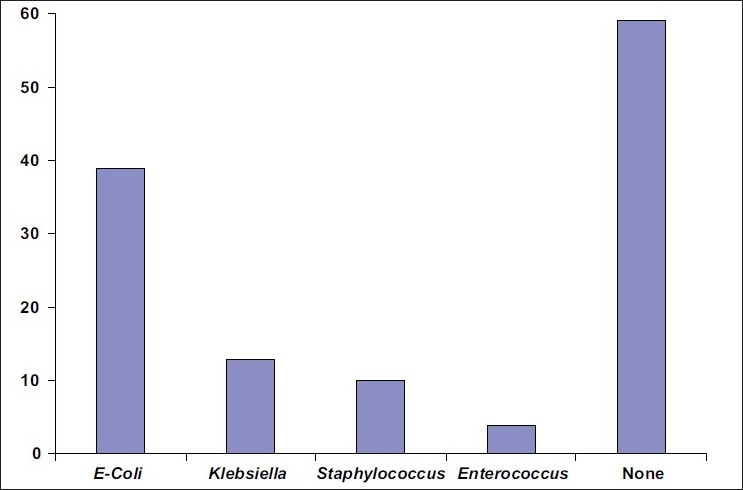
Number of patients infected with the various organisms identified by the Foley's tip culture

Only 28/125 (22.4%) of patients developed a symptomatic urinary tract infection during the period of follow up. All these patients also had a BCFC, which revealed the same organisms as the ones isolated form their urine culture. However, 38/125 (30.4%) patients developed just an asymptomatic colonization of their Foley's catheters during the period of catheterization. Therefore, majority of (66/125 or 52.8%) the patients developed a colonization of their catheters. Amongst all patients developing a BCFC, *E. coli* was the most frequent organism isolated (39/66, 59.1%). Other organisms isolated were *Klebsiella* (13/66), *Staphylococcus* (10/66) and *Enterococcus* (4/66). This is shown in Figure 1.

Of all the patients developing a BCFC, most (56/66 or 84.8%) were catheterized in the medical emergency as compared to only 10/66 (15.2%), who were catheterized in the wards. This was statistically significant. Other risk factors for developing a BCFC, as shown in Table 3, were: age >60 years, non-ambulatory functional status and duration of catheterization >3 days.

**Table 3:** Various risk factors associated with the development of a bacterial colonization on Foley's catheters

Parameter	Bacterial colonization on Foley's catheters		Odds ratio	Relative risk	Chi squared
				
	Yes	No
Age (yrs)					
<60	36	45	0.37	0.65	**6.45**
>60	30	14	0.16<OR<0.86	0.48<RR<0.89	***P*<0.05**
Sex					
Male	40	40	0.73	0.87	0.70
Female	26	19	0.33<OR<1.62	0.62<RR<1.21	*P*=0.40
Functional status					
Ambulatory	20	34	0.32	0.57	**9.48**
Non-ambulatory	46	25	0.14<OR<0.71	0.39<RR<0.84	***P*<0.01**
Mental status						
Alert	35	30	1.09	1.04	0.06
Impaired	31	29	0.51<OR<2.34	0.75<RR<1.45	*P*=0.81
Place of catheterization						
Emergency	56	36	3.58	2.01	**9.11**
Ward	10	23	1.42<OR<9.38	1.17<RR<3.46	***P*<0.01**
Duration of catheterization						
≤3 days	24	36	0.37	0.62	**7.59**
>3 days	42	23	0.17<OR<0.80	0.43<RR<0.89	***P*<0.01**

As for BCFC, CAUTI was also associated significantly with similar risk factors such as: higher age, alert mental status and a longer duration of catheterization. This is shown in Table 4.

**Table 4:** Various risk factors associated with the development of a catheter associated urinary tract infection

Parameter	Catheter associated urinary tract infection		Odds ratio	Relative risk	Chi squared
				
	Yes	No
Age (yrs)					
<60	13	68	0.37	0.47	**5.34**
>60	15	29	0.14<OR<0.95	0.25<RR<0.90	***P*<0.05**
Sex					
Male	19	61	1.25	1.19	0.23
Female	09	36	0.47<OR<3.35	0.59<RR<2.40	*P*=0.63
Functional status					
Ambulatory	10	44	0.67	0.73	0.82
Non-ambulatory	18	53	0.26<OR<1.73	0.37<RR<1.45	*P*=0.36
Mental status					
Alert	08	57	0.28	0.37	**7.93**
Impaired	20	40	0.10<OR<0.76	0.18<RR<0.77	***P*<0.01**
Place of catheterization					
Emergency	22	70	1.41	1.32	0.46
Ward	06	27	0.47<OR<4.39	0.58<RR<2.96	*P*=0.5
Duration of catheterization					
≤3 days	5	55	0.17	0.24	**13.13**
>3 days	23	42	0.05<OR<0.51	0.10<RR<0.58	***P*<0.01**

### Inappropriate catheterization

In our study, 36/125 (28.8%) patients were inappropriately catheterized. As shown in Table 2, patients with a CNS admitting diagnosis were most likely to be inappropriately catheterized (16/42, 38.1%) and those with a respiratory system diagnosis were least likely (2/11, 18.2%). The most frequent appropriate indication for catheterization was ‘altered sensorium’ (38/89, 42.7%) and the most frequent inappropriate reason for catheterization was urinary incontinence without significant skin breakdown (10/36, 27.8%). This is shown in Table 5.

**Table 5:** Various appropriate and inappropriate indications for catheterization along with their frequencies

Appropriate indications (89)	Inappropriate indications (36)
Urinary retention (8)	Transient incontinence (8)
Hemiparesis (20)	Immobility (8)
Altered sensorium (38)	Suspected retention (4)
Accurate monitoring of urinary output (15)	No explicit indication (6)
Chronically ill patients with urinary incontinence (4)	Urinary incontinence without significant skin breakdown (10)
Palliative care for terminally ill or severely impaired incontinent patients for whom bed and clothing changes are uncomfortable (4)	

The risk factors associated with inappropriate catheterization were female sex, ‘alert’ mental status and catheterization in the emergency [Table 6].

**Table 6:** Various risk factors for inappropriate catheterization

Parameter	Appropriately catheterized	Inappropriately catheterized	Odds ratio	Relative risk	Chi squared
Age (yrs)					
<60	61	20	1.74	1.18	1.89
>60	28	16	0.72<OR<4.14	0.92<RR<1.53	*P*=0.17
Sex					
Male	62	18	2.30	1.29	**4.30**
Female	27	18	0.96<OR<5.47	0.99<RR<1.69	***P*<0.05**
Functional status					
Ambulatory	34	20	0.49	0.81	3.15
Non-ambulatory	55	16	0.21<OR<1.17	0.64<RR<1.03	*P*=0.07
Mental status					
Alert	35	30	0.13	0.60	**19.89**
Impaired	54	6	0.04<OR<0.37	0.47<RR<0.76	***P*<0.01**
Place of catheterization					
Emergency	60	32	0.26	0.74	**5.03**
Ward	29	4	0.06<OR<0.87	0.61<RR<0.90	***P*<0.05**

Inappropriate catheterization was not significantly associated with development of a BCFC [Table 7].

**Table 7:** Association of appropriate catheterization with the development of a bacterial colonization on Foley's catheters

Indication for catheterization	Bacterial colonization on Foley's catheters		Odds ratio	Relative risk	*P* value
				
	Yes	No
Inappropriate	23	13	1.89	1.32	
Appropriate	43	46	0.8<OR<4.6	0.95<RR<1.83	*P*=0.11

### Duration of catheterization

The significant risk factors for longer duration of catheterization (>3 days) were age >60 years, non-ambulatory functional status and catheterization in the wards [Table 8].

**Table 8:** Various risk factors associated with duration of catheterization

Parameter	Duration of catheterization		Odds ratio parameter	Relative risk	Chi squared
				
	≤3 days	>3 days
Age (yrs)					
<60	45	36	2.42	1.63	**5.26**
>60	15	29	1.06<OR<5.60	1.03<RR<2.57	***P*<0.05**
Sex					
Male	40	40	1.25	1.13	0.36
Female	20	25	0.56<OR<2.78	0.76<RR<1.67	*P*=0.55
Functional status					
Ambulatory	36	18	3.92	1.97	**13.27**
Non-ambulatory	24	47	1.74<OR<8.90	1.35<RR<2.87	***P*<0.01**
Mental status					
Alert	34	31	1.43	1.21	1.01
Impaired	26	34	0.67<OR<3.09	0.83<RR<1.75	*P*=0.32
Place of catheterization					
Emergency	50	42	2.74	1.79	**5.63**
Ward	10	23	1.10<OR<7.16	1.03<RR<3.11	***P*<0.05**

## DISCUSSION

This study was conducted to analyze the practice of indwelling urinary catheters in medical wards. It has arrived at the conclusion that inappropriate catheterization is widely prevalent, even in a tertiary care referral center. Many patients who did not need a catheter in accordance with accepted indications were inappropriately catheterized and a large proportion of these subsequently went on to develop BCFC with many ending with a diagnosis of a CAUTI. The frequency of inappropriate catheterization in this study was lower (28.8%) as compared to previous studies, in which it has been estimated to be ranging from as 30-50%.[2,17,18] Nevertheless, this is still a substantial figure.

It was further found that around 52.8% developed a BCFC and 22.4% were diagnosed with a CAUTI, which is markedly higher than the previous study, where it was estimated to be around 11%.[1] These statistics emphasize the need for more stringent implementation of aseptic techniques while inserting a Foley's catheter and better infection control.

Urinary incontinence without significant skin breakdown emerged as the most frequent inappropriate indication for urinary catheterization. This is possibly attributable to the fact that catheterization in these patients may have been done to avoid inconvenience to the patient, their relatives and to the nursing staff. However, physicians should be extra vigilant while ordering catheterization for such patients.

The various statistically significant risk factors for inappropriate catheterization that have emerged from the study are female sex, ‘alert’ mental status and catheterization in the emergency. Doctors tend to be more liberal in catheterization of females possibly due to prevailing cultural factors relating to privacy issues in using a bedpan, ease of a catheterization and sometimes for patient preference. Female sex and non-ambulatory functional status has been shown to be a risk factor for inappropriate catheterization in a previous study.[19]

Alert mental status may not actually be a risk factor, but may have appeared as one due to a small sample size. However, a previous study by Raffaelle *et al*. has concluded that a ‘good state of consciousness’ is significantly associated with inappropriate use of indwelling urinary catheters.[18] Nevertheless, patients with an impaired mental status become more acceptable candidates for catheterization. This is because they are critically ill and may require accurate monitoring of their urine output. There may be associated paralysis in these patients and they may require palliative care for a terminal illness. All these factors make it more probable that when patients with an impaired mental status are catheterized; it is for an appropriate indication.

Catheterization in the emergency is more likely to be inappropriate as there is a heavy patient load and the decision to catheterize is spontaneous, sometimes without comprehensive evaluation of the real need for catheterization. Age was not a significant risk factor for inappropriate catheterization in this study which is in concordance with a previous study conducted by Munasinghe *et al*.[20]

The significant risk factors associated with BCFC are age >60 years, non-ambulatory functional status, catheterization in the emergency and longer duration (>3 days) of catheterization. CAUTI, which is the major morbidity arising out of catheterization of the urinary tract,[21,22] also had similar risk factors. Elderly patients are more susceptible to all infections and CAUTI is no exception.[2] Incidence of CAUTI in the elderly could be higher due to longer duration of catheterization, attributable to other co-morbidities present in them. Similar is the case with the non-ambulatory patients and those with an impaired mental status. A longer duration of catheterization would promote bacterial growth on the catheter surface and will also lead to a higher incidence of BCFC and CAUTI.

In our study, most patients were catheterized in the medical emergency, which appeared as a significant risk factor for inappropriate catheterization. However, when these patients were transferred to the wards most of these catheters were removed, accounting for the short mean duration of catheterization (4.3 days).

An interesting finding was that in spite of the shorter mean duration of catheterization in these patients (4.3 days) as compared to those catheterized in the wards (6.2 days), they had a higher incidence of BCFC and CAUTI. We attempted to investigate the reason behind this problem by verbally interviewing the residents managing the medical emergency after the data collection and analysis for the study was complete. This was done around two months after the study was complete. We randomly selected five residents out of a total of 51 residents in the department of medicine, who also manage the emergency.

The above findings were discussed with them and they were asked the following question: “In your opinion what is the probable reason for the higher incidence of CAUTI in patients catheterized in the emergency?” Four out of five residents interviewed stated that the reason for this was inadequate sterile precautions while inserting the catheter in the emergency due to time constraints. This significant finding calls for immediate re-education of the health care staff, their sensitization towards the appropriate use of IUTCs and for practicing sterile procedure techniques. A study by Gokula *et al*.[23] found that using a patient indication sheet could reduce the total as well as inappropriate catheterization in the emergency.

In this study, a higher proportion of inappropriately catheterized patients developed BCFC and CAUTI as compared to those who were appropriately catheterized, although this was not statistically significant. However, Topal *et al*.[24] have shown that inappropriate catheterization was linked to CAUTI and by subsequent reduction of use and duration of urinary catheters the incidence of CAUTI was significantly reduced (*P*<0.001). Hence, inappropriate use of indwelling urinary catheters appears to be a risk factor for CAUTI. Further studies are required to establish a definite relationship.

The various organisms isolated from the patients' catheter tips and urine culture showed Escherichia Coli to be the most frequent organism. Our results were similar to some old studies[25] but differ from others[26] where Pseudomonas and *Klebsiella* were the most frequent organisms isolated followed by *E Coli*. Other organisms isolated are also common pathogens of the urinary tract. Use of IUTCs acts as a significant reservoir of these organisms and may have a role in propagating other nosocomial infections.

### Limitations

The study has several limitations. As patients were included consecutively in the study and the sample collection was not random, therefore it may not be representative of the entire population. The study was uni-centric and results may not be universally applicable. Each patient was evaluated using the existing clinical scenario and interviews with the treating physicians/patients to decide if a criterion for IUTC placement was appropriate or inappropriate. There was no documentation in the patients' file regarding the IUTC insertion. However, we have attempted to address this concern by having two more reviewers for 25 randomly selected patients and obtained 100% agreement on the appropriateness of catheterization in the group. As only patients with a Foley's catheter were included in the study, the results may not apply to those with other types of catheters. Although the treating physicians were not informed about the purpose/details of the study, we cannot be sure whether or not they were influenced by the study and changed their decisions regarding indications and/or duration for catheterization. Our study design has not incorporated all possible risk factors for catheter associated urinary tract infections. However, we believe that these are not major limitations and would not have affected the results significantly.

### Strengths

Our study was different from previous studies, as we have evaluated the urinary tract infections in terms of not only symptomatic UTIs but also asymptomatic urinary tract colonization/infections. This is important as asymptomatic infections often go undetected and this may decrease the sense of accountability on the part of the physicians. Also, bacteria colonizing the catheter surface are a potential source of a CAUTI.[11] This study has highlighted the possible adverse practice of catheterization in the medical emergency, which has contributed to a majority of BCFC, CAUTI and inappropriate catheterization.

## CONCLUSION

Taking the results of the study into consideration, physicians should be sensitized to the need for carefully evaluating each patient and establishing a real requirement for catheterization before proceeding with the intervention. This is especially required for female patients as well as for those who are catheterized in the emergency. Also, strict aseptic precautions must be maintained while performing the intervention. Early decanulation, using a closed drainage system, smaller bore catheters, and adopting optimal hygienic techniques by health care workers are effective in minimizing the incidence of bacteriuria and ascending urinary infection.[27] In appropriate patients, suprapubic catheters, condom drainage systems and intermittent catheterization should be preferred to indwelling urethral catheterization.[9] Several new methods are being introduced to prevent CAUTI; for example, intravesical delivery of antibacterial gases into the urinary bladder.[28] Further clinical trials will elucidate whether this approach could have a place in clinical practice for management of CAUTI. As prevention is better than cure, CAUTI can be prevented by appropriate selection of patients and by strict aseptic techniques in using catheters.

## References

[1] Stamm AM, Coutinho MS (1999). Urinary tract infection associated with indwelling bladder catheter: incidence and risk factors. Rev Assoc Med Bras.

[2] Gokula RR, Hickner JA, Smith MA (2004). Inappropriate use of urinary catheters in elderly patients at a midwestern community teaching hospital. Am J Infect Control.

[3] Burke JP, Yeo TW, Mayhall CG (2004). Nosocomial urinary tract infection. Hospital epidemiology and infection control.

[4] Sedor J, Mulholland SG (1999). Hospital-acquired urinary tract infections associated with the indwelling catheter. Urol Clin North Am.

[5] Saint S (2000). Clinical and economic consequences of nosocomial catheter-related bacteriuria. Am J Infect Control.

[6] Saint S, Wiese J, Amory JK, Bernstein ML, Patel UD, Zemencuk JK (2000). Are physicians aware of which of their patients have indwelling urinary catheters?. Am J Med.

[7] Apisarnthanarak A, Thongphubeth K, Sirinvaravong S, Kitkangvan D, Yuekyen C, Warachan B (2007). Effectiveness of multifaceted hospitalwide quality improvement programs featuring an intervention to remove unnecessary urinary catheters at a tertiary care center in Thailand. Infect Control Hosp Epidemiol.

[8] Dalen DM, Zvonar RK, Jessamine PG (2005). An evaluation of the management of asymptomatic catheter-associated bacteriuria and candiduria at The Ottawa Hospital. Can J Infect Dis Med Microbiol.

[9] Tenke P, Kovacs B, Johansen TE, Matsumoto T, Tambyah PA, Naber KG (2008). European and Asian guidelines on management and prevention of catheter-associated urinary tract infections. Int J Antimicrob Agents.

[10] Ojha N (2008). Bacteriuria following Foley catheterization after gynecological and obstetrical surgery. Nepal J Obstet Gynaecol.

[11] Matsukawa M, Kunishima Y, Takahashi S, Takeyama K, Tsukamoto T (2005). Bacterial colonization on intraluminal surface of urethral catheter. Urology.

[12] Porth C (2007). Essentials of Pathophysiology: Concepts of Altered Health States.

[13] Kruse MJ (1986). Nursing the Neurological and Neurotrauma Patient.

[14] Warren JW, Andriole VT (1995). Catheter-associated urinary tract infections. Infectious disease clinics of North America: urinary tract infections.

[15] Slade N, Gillespie WA, Kunin CM (1985). Intermediate and long term catheter drainage. General principles of management in surgical, medical, geriatric, and neurological patients. Incontinence. The use of substitutes for catheter drainage. The urinary tract and the catheter: infection and other problems.

[16] Kunin CM, Slade N, Gillespie WA (1987). Care of the urinary catheter. Detection, prevention and management of urinary tract infections.

[17] Foxman B (2003). Epidemiology of urinary tract infections: incidence, morbidity, and economic costs. Dis Mon.

[18] Raffaele G, Bianco A, Aiello M, Pavia M (2008). Appropriateness of use of indwelling urinary tract catheters in hospitalized patients in Italy. Infect Control Hosp Epidemiol.

[19] Apisarnthanarak A, Rutjanawech S, Wichansawakun S, Ratanabunjerdkul H, Patthranitima P, Thongphubeth K (2007). Initial inappropriate urinary catheters use in a tertiary-care center: incidence, risk factors, and outcomes. Am J Infect Control.

[20] Munasinghe RL, Yazdani H, Siddique M, Hafeez W (2001). Appropriateness of use of indwelling urinary catheters in patients admitted to the medical service. Infect Control Hosp Epidemiol.

[21] Adukauskiene D, Cicinskaite I, Vitkauskiene A, Macas A, Tamosiūnas R, Kinderyte A (2006). Hospital-acquired urinary tract infections. Medicina (Kaunas).

[22] Danchaivijitr S, Dhiraputra C, Cherdrungsi R, Jintanothaitavorn D, Srihapol N (2005). Catheter-associated urinary tract infection. J Med Assoc Thai.

[23] Gokula RM, Smith MA, Hickner J (2007). Emergency room staff education and use of a urinary catheter indication sheet improves appropriate use of foley catheters. Am J Infect Control.

[24] Topal J, Conklin S, Camp K, Morris V, Balcezak T, Herbert P (2005). Prevention of nosocomial catheter-associated urinary tract infections through computerized feedback to physicians and a nurse-directed protocol. Am J Med Qual.

[25] Nicolle LE (2005). Catheter-related urinary tract infection. Drugs Aging.

[26] Cetin BD, Hasman H, Ozcan N, Gündüz A, Harmankaya O, Seber E (2005). Epidemiology and etiology of catheter-related nosocomial infections in a Turkish hospital. Infez Med.

[27] Ramakrishnan K, Mold JW (2005). Urinary Catheters: a review. J Fam Pract.

[28] Carlsson S, Weitzberg E, Wiklund P, Lundberg JO (2005). Intravesical nitric oxide delivery for prevention of catheter-associated urinary tract infections. Antimicrob Agents Chemother.

